# Is the European Crohn’s and Colitis organisation (ECCO) e-guide an acceptable and feasible tool for increasing gastroenterologists’ guideline adherence? A mixed methods evaluation

**DOI:** 10.1186/s12909-024-05540-w

**Published:** 2024-05-13

**Authors:** Ria Kanazaki, Ben Smith, Stella Bu, Afaf Girgis, Susan J Connor

**Affiliations:** 1 South West Sydney Clinical Campuses, Faculty of Medicine & Health Sciences, University of New South Wales, Sydney, NSW Australia; 2 Ingham Institute for Applied Medical Research, Sydney, Australia; 3 The Daffodil Centre, The University of Sydney, A Joint Venture with Cancer Council NSW, Sydney, NSW Australia; 4 Department of Gastroenterology and Hepatology, Liverpool Hospital, Sydney, Australia

**Keywords:** eHealth, Clinical practice guidelines, Guideline adherence, Inflammatory bowel disease

## Abstract

**Background and aims:**

Management of inflammatory bowel disease is constantly evolving, increasing the importance for gastroenterologists to keep up to date with guidelines. Traditional implementation strategies have had only small positive impacts on clinical practice. eHealth strategies such as the European Crohn’s and Colitis Organisation e-guide may be beneficial for clinician decision making in keeping with guidelines. The aim of this study was to evaluate the feasibility and acceptability of the e-guide.

**Methods:**

A mixed methods approach was used to evaluate feasibility and acceptability. Cognitive (think-aloud) interviews were conducted with Australian gastroenterologists while using the e-guide. Two clinical scenarios were developed to allow evaluation of various aspects of the e-guide. Content analysis was applied to the qualitative interview data and descriptive analysis to the quantitative and observational data.

**Results:**

Seventeen participants completed the study. Data saturation were reached. The ECCO e-guide was largely feasible and acceptable, as demonstrated by most clinical questions answered correctly, 87% reaching the answer within 3 min, and most feeling it was useful, would be beneficial to their practice and would use it again. Issues raised included difficulties with website navigation, layout of the e-guide and difficulties with access (network firewalls, paid subscription required).

**Conclusions:**

The ECCO e-guide is largely acceptable and feasible for gastroenterologists to use. Aspects of the e-guide could be modified to improve user experience. This study highlights the importance of engaging end-users in the development and evaluation of clinician educational tools.

**Supplementary Information:**

The online version contains supplementary material available at 10.1186/s12909-024-05540-w.

## Background

The management of inflammatory bowel disease (IBD) has evolved over the last decade, making it critical that gastroenterologists keep up to date with the latest clinical practice guidelines (CPGs). CPGs are informed by systematic reviews of the highest levels of evidence available to support beneficial clinical practices and can reduce unwanted variations and improve patient care outcomes [1, 2]. Despite availability of CPGs on optimal IBD care, suboptimal adherence persists in acute, chronic, and preventive healthcare settings [3–6].

Traditionally, CPGs have been disseminated via distribution of educational materials and educational meetings, which have demonstrated small positive impacts on desired clinical practices [7, 8]. Gastroenterologists currently access CPGs through journal publications or gastroenterology organisation websites. A recent study of gastroenterologists in Australia reported that despite most participants reporting confidence in the European Crohn’s and Colitis Organisation (ECCO) guidelines, less than half referred to them regularly [9]. Reported barriers to gastroenterologists’ guideline adherence include: time constraints, limited familiarity with guideline specifics due to the volume of information, length of CPGs making them impractical to use, and previous negative experiences (e.g. adverse patient outcomes) [9, 10]. More effective approaches to disseminating evidence-based guidelines are required to achieve greater impact on practice.

As a developing field, eHealth facilitates the delivery of health services and information through the internet and related technologies. eHealth is increasingly being used for delivering continuing medical education in healthcare settings. The use of eHealth can overcome geographical barriers and time constraints, offering a more flexible and accessible alternative to traditional learning. It also allows for interactive learning resources that can be easily updated in line with new evidence [11].

One form of eHealth intervention is electronic clinical decision support tools, often used during a patient consultation. They provide patient-specific assessments or recommendations to assist clinicians’ decision-making, with the aim of improving care quality and maintaining patient safety [12]. Examples include alerts, reminders and search tools to aid procurement of patient-specific evidence-based information. Use of clinical decision support tools can enhance clinical outcomes, including reducing morbidity, as they facilitate reduced variation in care and promote CPG adherence [13–15]. Studies of clinical decision support systems in medical specialties other than gastroenterology have demonstrated improved healthcare process measures including performing preventative services, ordering investigations, and prescribing medical therapies [14, 16].

**Fig. 1:**
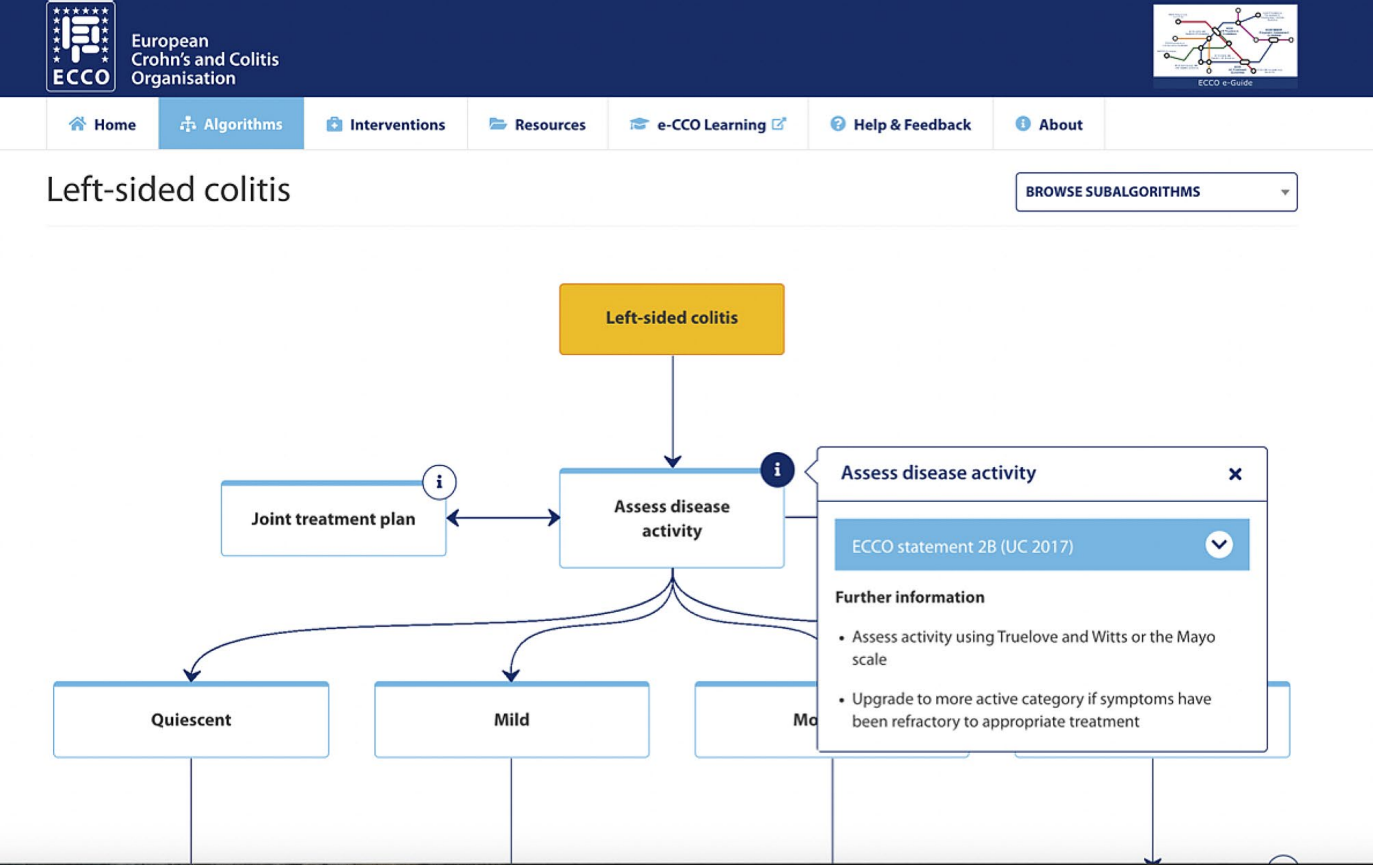
Screenshot of the ECCO e-guide algorithm for the management of left-sided colitis by disease activity (http://www.e-guide.ecco-ibd.eu/algorithm/extensive-colitis,, accessed 21/5/23)

Current evidence for the role of eHealth in IBD CPG adherence is limited; no studies have assessed the efficacy of eHealth interventions targeting clinicians in this area. The ECCO e-Guide (http://www.e-guide.ecco-ibd.eu/), hereafter referred to as the e-guide, is a freely accessible eHealth resource for IBD healthcare professionals. The e-guide visualises the ECCO guidelines as algorithms to provide a practical tool to aid clinician decision-making regarding IBD patient management (see Fig. 1 for an example). To our knowledge, the e-guide has not been formally evaluated to date. This study aimed to test the acceptability, including user experience and attitudes towards the intervention, and feasibility, including practicality and implementation issues, of the e-guide to gastroenterologists.

## Methods

### Study design and setting

A mixed methods study was undertaken with clinicians working in the field of gastroenterology in Australia. Cognitive (i.e., think-aloud) interviews were conducted online using Zoom (San Jose, USA), enabling gastroenterologists to participate from anywhere in Australia. Interviews were conducted between October 2019 and January 2020. The COnsolidated criteria for REporting Qualitative research (COREQ) checklist were used for reporting (supplementary material – appendix A). Participants completed the interviews on computers at a location of their choosing.

### Sample

The study aimed to recruit a purposive sample of 20 clinicians of varying years of clinical experience and IBD patient volumes. Interview participants were a sub-group of gastroenterologists who had participated in a previous survey and consented to be contacted for this interview [17]. To gain diverse perspectives, the purposive sample was chosen to include participants with varying degrees of experience using the e-guide.

### Procedure

Two clinical scenarios were developed (Supplementary data - Appendix B), which required clinicians to navigate the e-guide to answer clinical questions. The first scenario was a new diagnosis of stricturing ileal Crohn’s disease, and the second scenario was an ulcerative colitis case with increasing disease activity on a 5-aminosalicylate. The scenarios were developed and piloted with an IBD expert (SC) and designed to allow evaluation of various aspects of the e-guide. The acceptability and feasibility of the e-guide as an aid to promote guideline adherence was evaluated via cognitive interviewing, a method of exploring an individual’s thought processes, which is increasingly being used in the development and evaluation of education materials [18]. In one-on-one interviews, respondents described their thoughts whilst answering questions presented to them while using the e-guide. An example question from scenario two (Supplementary data - Appendix B) is “You would like to plan what monitoring the patient will require for his thiopurine. What thiopurine monitoring is suggested for the next 3 months?” Commonly referred to as “think-aloud” interviewing, cognitive interviews allow researchers to understand how individuals perceive and interpret information and to identify any potential problems in navigating and understanding educational materials [19].

At the completion of the two clinical scenarios, participants were asked several questions by the interviewer to further explore the acceptability and feasibility of the e-guide. Acceptability was evaluated in the interview by focusing on the areas of: user experience, affective attitude towards the intervention, willingness to participate in the intervention, and burden [20]. Feasibility was evaluated in the interview by focusing on issues related to implementation, practicality, and adaptation of the e-guide [21]. Repeat interviews were not conducted.

Interviews were conducted by a single interviewer (RK) and were audio and screen recorded to understand participants’ thought processes whilst capturing how they navigated the e-guide. The interviewer, a female Australian gastroenterologist (MD), was known to all participants. The participant and interviewer were the only persons present. Prior to the cognitive interview, each participant completed a practice think aloud task whilst comparing images from the website: spot the difference (http://www.spotthedifference.com/). Participants were not expected to have prior knowledge of the e-guide. A link to the e-guide home page was provided to participants via email, which they accessed using a desktop computer, as was recommended at the time. During the cognitive interview, participants were encouraged to think aloud. The interviewer remaining largely silent, unless prompting was required to encourage them to think aloud, or when they had difficulties reaching the correct answer in which case the question was repeated with the focus of the question emphasised. Further, notes were made during interview analysis to record additional observations about pathways the participants took and problems experienced whilst using the e-guide. Interview transcripts were not returned to participants for comment.

### Data analysis

Qualitative and quantitative data were collected to evaluate the acceptability and feasibility of the e-guide. Qualitative analysis of interview audio recordings was performed, with one researcher experienced in qualitative analysis (SB) reviewing transcripts and applying content analysis to identify relevant themes [22]. A second researcher (RK) coded a sub-set of five interviews to ensure that codes generated covered all relevant aspects of the data. Using Microsoft Excel codes were then collated into potential themes, with discussion between the two coders to ensure theme content was consistent and distinct from other themes. The coding tree is presented in supplementary material - appendix C. Quantitative data collected from the interview audio and video recordings including time taken to reach the answer, number of clicks per answer, number of prompts required and what item was first clicked was collected manually by the researcher and analysed descriptively. Observational data from interview audio and video recordings regarding any problems the participants experienced whilst navigating the site were also collated and described.

## Results

### Study sample

Seventeen participants completed interviews prior to the e-guide website undergoing development changes. These included changes to the website layout and management algorithms, and modifications to the resource section including removal of video resources and patient materials. The decision was made to close recruitment as the same clinical scenarios could not be applied for future participants. Data saturation were also achieved after 17 interviews with no new themes identified. Of the participating clinicians, 13 were male gastroenterologists, nine saw a high volume of IBD patients (> five patients per week), eight were aware of the e-guide, only one referred to it often (see Table 1). The interviews took between 21 and 57 min (mean 32 min) to complete. Twelve participants were audio and video recorded, and 5 were audio recorded only due to technical issues. All participants who had agreed to participate completed the study.

**Table 1:** Participant demographics

Participant ID	Male/Female	E-guide aware	IBD patient load*	Years in practice
1	Male	No	High	< 5 years
2	Male	No	High	< 5 years
3	Male	No	High	in training
4	Male	No	High	< 5 years
5	Male	Yes	Low	< 5 years
6	Male	No	Low	6–10 years
7	Female	Yes	Low	in training
8	Female	Yes	High	< 5 years
9	Female	No	Low	6–10 years
10	Male	Yes	High	< 5 years
11	Male	No	Low	< 5 years
12	Male	Yes	High	6–10 years
13	Male	No	High	11–20 years
14	Male	No	Low	< 5 years
15	Male	No	Low	< 5 years
16	Male	Yes	Low	< 5 years
17	Female	Yes	Low	in training

*High = > 5 pts/week, Low = < 5 pts/week

### Feasibility evaluation

#### Correct answer reached

Across the two IBD patient scenarios (Supplementary data - Appendix B) there were ten clinical questions answered by 17 participants (170 questions total). 167/170 questions were correctly answered. Three different participants incorrectly answered the same question from the second scenario asking, “what medical therapy is recommended next?” for a patient with a flare of ulcerative colitis on a maintenance dose of oral 5-aminosalicylate(5-ASA) alone. The information to answer this question required participants to click on the information box associated with the part of the algorithm they were at, ‘topical and oral mesalazine’, whereas these three participants continued to follow the algorithm to the next step which was ‘add oral steroids’. Dose optimisation of 5-ASA was not included as a step in the algorithm, but was located within information linked to it, which is why it was missed.

#### Time and path taken to reach the answer

The participants arrived at the answers within a range of 1–612 s (median 73 s), with 43%, 75% and 87% reaching the answer within 1,2, and 3 min respectively. This is described for each question in Tables 2a and 2b. Three participants were unable to reach the correct answer despite prompting by the interviewer for the same question as discussed above. Eight of the ten questions had one participant who took notably longer than the rest of the group to reach the answer. The predominant reason for longer times to reach the answer was uncertainty regarding where information was located resulting in clicking through every tab on the homepage, and participants manually scrolling through the resource section which contained a variety of resources including patient information, videos, endoscopic score calculators, and journal articles. There were a variety of paths taken to reach the answer demonstrated by different initial clicks (see Table 2a and 2b). The layout was reported as unhelpful by some, for example the ulcerative colitis algorithm was not completely visualised on one screen which resulted in information being overlooked, leading to a longer and less direct path to the answer taken,

**Table 2:** a. Quantitative interview data and observational comments for Crohn’s Disease Case

Question (summary)	Number of clicks for most common starting point (number of different starting points for initial click)	Time to click answer (sec) Median (Range)	Number of clicks to answer** Median (Range)	Number of prompts Median (Range)	Comments
Q1 (Follow Ileal stricture management algorithm)	7 (7)	99 (41–258)	3 (2–10)	0 (0–3)	
Q2 (Find video demonstration)	4 (11)	108 (10–429)	10 (3–18)	0 (0–2)	Third longest time to reach the answer. Photos and videos of the same procedure not linked together in ‘endoscopic procedures’ Sect. 12/17 participants found photos first. Illogical placement of video. Feedback - video unhelpful without audio - has presumed knowledge.
Q3 (Find patient guidelines)	10 (5)	26 (6-183)	3 (2–5)	0	

**Four participants are not included due to no video data

**Table 2:** b. Quantitative interview data and observational comments for Ulcerative Colitis Case

Scenario/Question	Number of different starting points for initial click (clicks for most common starting point)	Time to click answer (sec) Median (Range)	Number of clicks to answer** Median (Range)	Number of prompts Median (Range)	Comments
Q4 (Find infection checklist)	3 (10)	43 (1-164)	2 (1–7)	0 (0–1)	
Q5 (Find and use mayo score calculator)	7 (6)	73 (26–336)	7 (2–35)	0 (0–1)	Higher range of prompts needed. Found mayo score data sheets but not the calculator.
Q6 (Follow ulcerative colitis algorithm)	10 (5)	78 (36–212)	2 (2–14)	0 (0–4)	Higher range of prompts needed due to the answer being within the information icon and not clearly visible. Three participants did not reach the correct answer.
Q7 (Ulcerative colitis algorithm for treatment escalation)	17 (1)	5 (1–19)	0 (0)	0 (0)	
Q8 (Steroid refractory algorithm)	6 (5)	158 (31–437)	8 (1–42)	1 (0–4)	Second longest time to reach the answer. Due to the algorithm layout not entirely visible on one screen (needed to scroll up), missing the steroid refractory algorithm. Disagreed with definition of steroid refractory. Disagreed with recommendation due to Pharmaceutical Benefits Scheme.
Q9 (Thiopurine monitoring)	4 (9)	84 (34–226)	8 (4–19)	0 (0–1)	High clicks range. Most found it straightforward. Hard to find information for some participants. Four people used the search function in resources section with no results.
Q10 (Relapse risk with stopping therapy)	6 (8)	100 (35–612)	6 (2–40)	0 (0–4)	Longest time to get to the answer. Difficult to search. Evidence to support statement was a study in ulcerative colitis and not Crohn’s but is not mentioned. Questioned accuracy of information. Unhelpful layout.

**Four participants are not included due to no video data

#### Implementation and practicality issues

Whilst navigating the e-guide website, some participants noted that there were some resources that only paid ECCO members could access, for example the extra-intestinal manifestations part of the new presentation of Crohn’s disease algorithm. Requiring a paid subscription to gain access to information may potentially deter future clinicians from using a tool like this.

Participants who were interviewed on computers accessing the internet via the public hospital network experienced difficulties accessing some areas of the website due to firewalls. For example, this was an issue when using links provided in the e-guide which opened a page to an external website, such as the online Mayo calculator which participants were asked to use to calculate a partial Mayo score. Whilst this issue was limited to participants accessing the e-guide from within a public hospital, it is an important consideration, as many IBD patients are seen in clinics within public hospitals and their clinicians could benefit from accessing a clinical decision support tool like this.

Some participants disagreed with the treatment recommendations in the algorithms, as they did not comply with prescribing limitations within Australia, despite being consistent with ECCO guidelines. For example, the e-guide recommended a patient with extensive ulcerative colitis on 5-ASA therapy with moderate to severe disease activity have treatment escalated directly to biologic therapy. In Australia, this would not be possible as the pharmaceutical benefits scheme (PBS) would require three months of thiopurine prior to approving biologic therapy, “these are European guidelines. So obviously doesn’t have to come to PBS recommendations but for an Australian looking at this…I can’t go to anti TNF (tumour necrosis factor)” (ID1).

### Acceptability Evaluation

#### Accessibility to the e-guide

Having easily accessible information was considered important for participants to use a clinical decision support tool, like the e-guide, frequently. Most participants already use a computer or smartphone to access information during consultations, which they felt would make it easy for them to access in the future. Several participants also felt that if the e-guide was available via a smartphone app this would further improve its usability as they were more likely to use an app as opposed to a website, “I think if you can make it in an app form, on my phone, it would be a lot better” (ID11).

Participants were asked to access the patient guidelines on the e-guide site which required them to enter their email address for the document to begin downloading to their device. This did cause some concern to participants as they were unsure the purpose of obtaining their information, “I don’t really like giving out my email address. Why do they ask…? I’ll know if my email starts getting bombarded” (ID2).

#### Familiarity with the e-guide

Most participants were unaware of the e-guide or had little experience using the website (see Table 1). Some felt that as a first-time user, it was “hard to get your head around….not intuitive” (ID9) and “if you are not familiar…trying to use it for the first time, then you could spend like I did 10 minutes trying to find it” (ID1).

All the participants felt that with increased exposure to the website, it would become easier to use, with information found more quickly, “once you get an idea of it…you can incorporate it into the visit and search fairly quickly” (ID1) and thinking “that there’s probably a learning curve, and if you use it more frequently, you’ll probably find what you’re looking for quite easily” (ID7).

#### Website navigation

Some of the participants found the e-guide easy to use - “for most things I got to what I wanted fairly quickly” (ID11). The majority however, reported some difficulties navigating the website. Participants thought it was somewhat “clunky” (ID10). When clicking ‘back’ to the previous homepage it “might exit you out of the whole guide and you have to start back into it” (ID14).

One participant noted that the homepage contained a link to a tour of the website, however felt that “most people wouldn’t” access it (ID1).

The questions which participants found most difficult, took the longest time to answer and required the most prompts, were primarily due to issues navigating the website. For example in the ulcerative colitis scenario, the patient continued to have disease activity with the management algorithm followed. Participants were unsure where to go on the algorithm as the final instruction at the bottom of the page was ‘treatment based on activity/extent’, whereas the next step for their management was at the top of the page which was often overlooked.

#### Layout preference

The information needed to answer some questions was not located in a logical place for many participants. For example, participants were asked to view a video of an anastomotic dilatation, with the majority incorrectly first looking in the ‘endoscopic procedures’ section located under the ‘interventions’ tab on the home page. The video was subsequently located under the ‘resources’ section with one participant reporting “I find some of the naming of the tabs are a bit odd. I’m not sure how does disease info is different from… resources and the same with interventions” (ID11). Another participant also agreed that the naming of the headings made locating information difficult, “I think some of the titles don’t quite match up to what you would expect them to have” (ID16).

The absence of a search function tool was highlighted by several participants as a feature which would improve the e-guide’s usability, “what we’re looking for in a guide like this is a quick jump to that information” (ID5). Difficulties locating information were thought to impact on its use in practice, as clinicians are already under time pressure in a busy clinical setting. Participant views supporting this include - “I think because it’s not as intuitive and not that easy to navigate for certain things, you might not be able to use it in practice because it takes time to find those things” (ID16); and “I don’t know why it’s not there ‘cause it seems like a no-brainer that you would have a keyword search” (ID9).

#### Content satisfaction

Most agreed with the clinical recommendations provided within the e-guide. The algorithms were thought to be helpful in guiding management, however some felt they were too simplified, structured and “prescriptive” (ID14). The algorithms were unable to account for patient nuances, which some thought could be harmful, “it does worry me that people try to follow these strictly and I think you can make the wrong decisions and it’s not as simple” (ID12). For example, in the scenario describing a new diagnosis of stricturing Crohn’s disease with a symptomatic ileal stricture > 5 cm, the algorithm indicates the next step is surgical management, but some participants felt the decision was not so straightforward, “there should have been some discussion of medical therapy…they should have a box saying an MDT(multidisciplinary team) type discussion and consideration of medical and surgical therapy in combination” (ID14).

The ulcerative colitis algorithm (second scenario) categorised patients by their level of disease activity (e.g. mild, moderate, severe) to guide management decisions which some clinicians found challenging to follow. They felt that categorising by disease severity can be “arbitrary” and that the patient “doesn’t fit one category” (ID5). There also may be “some variation in different clinicians’ mind as to whether to call something moderate or severe” (ID13). Including information from guidelines on how to categorise disease severity may be useful in this setting.

Some participants did not agree with the definition of steroid refractory disease in the ulcerative colitis algorithm, which caused 8/17 participants to initially answer the question incorrectly, requiring prompting by the interviewer, “Yeah, I definitely didn’t agree with that…that’s something I need to look up. It doesn’t really make intuitive sense if you stop steroids two-and-a-half months ago and you flared and then for you to be classified as steroid refractory” (ID10). Another participant was able to gain an understanding of the definition through the information box attached to the algorithm, “the info bubbles were helpful giving information about what they meant by a term like steroid refectory, for instance” (ID14).

Some felt that a resource such as this may be most beneficial to a general gastroenterologist or someone less experienced in IBD management. “I think for someone who doesn’t treat Crohn’s or ulcerative colitis often, I think it gives a nice step by step approach. Especially if you’re not as comfortable or don’t have that much experience” (ID1). Clinicians with IBD experience also found the e-guide content useful and thought they would use it again, for example the pregnancy recommendations, which some were not comfortable with managing. However many were also unaware of the e-guide, “I haven’t heard many people talking about it” (ID4).

#### Perceived benefits of the e-guide

Most participants thought that the e-guide would be beneficial to their clinical practice and would use it again. Benefits included providing a systematic approach to patient management “they kind of step you through everything you need to do” (ID7); and also assisting with patient discussion, “I like the fact that…there are some illustrative diagrams there on risks of thiopurine…I think having some easily accessible information that you can show your patients at the time and having the resource to print guides for them” (ID12).

#### Usefulness and ease

The information provided in the management algorithms also contained the corresponding ECCO guideline key recommendations, which many found useful, “if I wanted to know more about a particular box, there’s that information icon which takes me to the corresponding ECCO statement which is good” (ID11). Concise and simple statements were preferred to allow for easy readability of the e-guide in a busy clinical setting, however some participants wanted to be provided with the evidence for the key recommendations, “what is the evidence behind it? Rather than me having to search for it” (ID5).

In the first scenario, managing a new diagnosis of stricturing disease Crohn’s disease, participants were asked to access a video resource of an anastomotic dilatation for their own future reference. Most felt the video was unhelpful and that additional information was needed. The video did not contain audio instructions or written information to explain the technical aspects of the procedure. They felt that if the video was updated to include this information that the resource would be useful to them, “I think the video needs some sort of orientation information…give you information on what is going on because the images themselves are not giving the whole picture” (ID9).

## Discussion

The ECCO e-guide is the first clinical decision support tool developed for IBD management that provides guidelines in an alternative format, as algorithms, further to providing other evidence-based information. To our knowledge, this study is the first formal evaluation of the e-guide. Overall, the results suggest that the e-guide is largely feasible, with the correct answer reached within a short time frame by most participants, and acceptable to gastroenterologists, according to qualitative feedback indicating that the majority found the e-guide useful, agreed with its content and found it easy to use with repeated use. Potential modifications to the e-guide were identified, particularly relating to website navigation and layout, that could further enhance its acceptability and feasibility.

Most participants found the e-guide feasible to use, as evidenced 75% reaching the correct answer within two minutes. Being able to find the relevant information quickly has been identified as an important factor for gastroenterologists when accessing evidence-based information online as they are often time poor [10]. A systematic review of factors that impact the success of guideline-based clinical decision support tools also identified time constraints as a common barrier to clinicians’ uptake of them and that in order to be successful they should limit the impact on clinicians workflow, but be integral to their practice [23].

All participants accessed the e-guide on their desktop computer for the interview, however many suggested a smartphone application of the e-guide would increase their use of it. This is consistent with the findings of a previous study where smartphone applications was a suggested strategy to increase gastroenterologists’ access to evidence-based guidelines [10]. Medical education has evolved to incorporate smartphone applications which have been used by clinicians for example as medical calculators, clinical-decision support tools and diagnostic algorithms which have been found to be effective tools for enhancing knowledge and skills [24, 25]. Smartphone applications for clinicians’ provide several benefits including increased access to point-of -care tools which support better clinical decision-making and can improve patient outcomes [26]. We note that the latest iteration of the e-guide is now smartphone compatible.

Most clinicians interviewed had little knowledge or previous experience with the e-guide, despite ECCO being the most commonly referred to guideline for IBD management in Australia [17]. Whilst many participants initially found the e-guide difficult to navigate, this improved with increased familiarity whilst they completed the tasks for both scenarios over the course of the interview. We acknowledge that ECCO would not have specifically marketed the e-guide in Australia and are unaware of the current uptake of the e-guide in Europe. The relationship between promotion of educational tools and clinician uptake is not well understood, although limited awareness of clinical practice guidelines has previously been identified as a barrier to uptake [9, 27]. Promotion of educational tools is an important consideration when developing interventions, to enhance the potential impact on clinicians’ knowledge and behaviour.

Factors underlying the acceptability and efficacy of online clinician education interventions are not well known [28]. The cognitive interviews highlighted areas where clinicians experienced difficulties with website navigation which increased the time taken to find the relevant information. The lack of an adequate search function was highlighted by several participants as an issue, as well as the layout not being ‘intuitive’ with unclear headings to indicate where specific information would be found. Difficulties with navigation are likely to impact clinician uptake due to time constraints, a previously identified barrier for guideline adherence [9, 10]. Providing clear navigation information, particularly in a management algorithm, to guide clinicians on appropriate next steps would be helpful alongside website homepage headings that are straightforward and clearly differentiate each section. Clinicians are time poor and for an intervention to be successfully taken up it will need to be referred to quickly and easily.

Disagreement with guideline recommendations is a commonly reported barrier to guideline adherence which can be due to lack of applicability to a given scenario and lack of agreement with the evidence [27, 29]. Overall, participants agreed with e-guide recommendations. However, in the UC management algorithm there was disagreement with the recommendation to escalate directly from 5-ASA to anti-TNF or vedolizumab due to prescribing restrictions in Australia, which have previously been reported to be a barrier for adherence to IBD guidelines [10]. An intervention that does not comply with the local regulations may impact its clinician uptake. We acknowledge that the e-guide was developed with the European gastroenterology audience in mind and was not intended to be an educational tool in Australia. Educational interventions need to be tailored for their target audience working in different contexts to be most effective.

The majority were satisfied with the e-guide content and reported they would use it again. Participants found the e-guide’s step-by-step approach of presenting guidelines in an algorithm useful for guiding their patient management decisions. The content was considered beneficial to a gastroenterologist with a specific IBD interest, but more so for the general gastroenterologist who would have less IBD knowledge and clinical experience. Studies have shown that there is variation in patient care given by general gastroenterologists compared to those with an IBD interest, with general gastroenterologists more frequently deviating from guidelines [3, 10, 30]. The algorithms alone may be beneficial for a general gastroenterologist however more detailed information, provided by the links within the algorithm, may be more beneficial for those with a specific interest in IBD. The acceptability of an intervention may be enhanced by providing different levels of information to target a wider gastroenterology audience that manage IBD patients.

Evaluation is an essential component of developing effective health education interventions with benefits including understanding clinicians’ educational needs, identifying strengths and weaknesses of interventions, and assessing intervention outcomes [31–33]. In IBD many educational interventions have been developed by IBD groups and industry sponsors, however formal evaluation has been limited. The “IBD live” webcast program, implemented in the Unites States using telemedicine, is the only IBD educational intervention we are aware of that assessed its feasibility with participants reporting that it resulted in increased implementation of new management strategies, increased use of evidence-based guidelines and improved communication with patients and their families [34, 35].

Our study has some limitations. The results reflect the experience of Australian gastroenterologists using the e-guide, although the overall themes are likely to be relevant to gastroenterologists in other developed countries. There are however, some differences, predominantly related to practice under a different healthcare system (e.g. variation in prescribing restrictions). Another limitation is that the e-guide has undergone several updates since this study was conducted including a modified resource section and the website is now smartphone compatible, however the overall layout and format of guidelines being presented as algorithms has not changed. Our results are still likely to be relevant, as the overarching themes discussed below are not specific to the changes, but to the overall usability of the e-guide. Finally, it was only possible to analyse audio recorded cognitive interview data for five of the 17 participants, as video data were not available due to technical issues. Potentially informative data may have been missed due to the lack of video, however as the participants were thinking aloud this would have been minimised.

## Conclusion

This study shows that the ECCO e-guide is largely acceptable and feasible for gastroenterologists to use. Themes from the study identified aspects of the e-guide that could be modified to improve their experience including website navigation and layout, and highlighted the importance of engaging end-users in the development and evaluation of clinical decision support tools and clinician education. Further refinements to the e-guide based on user feedback with further evaluation in the European context would be useful. Future studies to assess the impact of the e-guide on clinician knowledge of CPGs and practice behaviour would be valuable in addition to a comparative analysis with other CPG implementation strategies. An evaluation of the relationship between awareness of the e-guide and adoption of its recommendations would also be useful.

### Electronic supplementary material

Below is the link to the electronic supplementary material.

Supplementary Material 1

Supplementary Material 2

## Data Availability

The datasets generated during and/or analysed during the current study are available from the corresponding author on reasonable request..

## Abbreviations

IBD: Inflammatory bowel disease
CPG: Clinical practice guidelines
ECCO: European Crohn’s and Colitis Organisation
5- ASA: 5-aminosalicylate
PBS: Pharmaceutical benefits scheme
TNF: Tumour necrosis factors
MDT: Multidisciplinary team
