# Supplementary material for: Is the European Crohn’s and Colitis organisation (ECCO) e-guide an acceptable and feasible tool for increasing gastroenterologists’ guideline adherence? A mixed methods evaluation
Source: BMC Med Educ. 2024 May 13;24:529. doi: 10.1186/s12909-024-05540-w (PMC11092016; doi:10.1186/s12909-024-05540-w)
Supplement: Supplementary file 1 — Supplementary Material 1 [file 12909_2024_5540_MOESM1_ESM.docx]

**Supplementary Data**

**Appendix B**

**Scenario 1**

*A 40 year-old woman is referred to you by her GP with a suspected new diagnosis of Crohn’s disease. She reports a several month history of abdominal pain, non-bloody diarrhoea, and weight loss.*

*Colonoscopy found an ileocaecal valve that was ulcerated and inflamed. The scope was inserted into the TI before reaching an inflammatory stricture which the scope was unable to pass. The remainder of the colon was normal.*

*MRI enterography found an ileal stricture. The stricture is 10cm from the ileocaecal valve and measures 15cm in length. There is minor proximal bowel dilatation.*

**Questions**

1. **What treatment is recommended for the ileal stricture?**

*She undergoes a surgical resection and some time later develops an anastomotic stricture. You want to review how to perform an anastomotic dilatation.*

1. **Can you find a video demonstration of an anastomotic dilatation in the e-guide?**

*Your patient is asking you for any patient guidelines they can take home with them.*

1. **Can you find the Crohn’s Disease patient guidelines for your patient?**

**Scenario 2**

*A 35-year-old man who was diagnosed with ulcerative colitis 5 years ago comes to clinic. He had pancolitis at diagnosis and has been maintained on mesalamine 2g daily for the past few years. He now presents with increased stool frequency (3 more than usual per day), rectal bleeding on most bowel motions. He denies any recent antibiotic use. He has not been screened for opportunistic infections.*

1. **You would like to review the opportunistic infections checklist prior to any escalation of her therapy. Can you find this?**

*You assess the disease activity by performing a colonoscopy. There was moderate disease activity of the entire colon with marked erythema, loss of vascular pattern and erosions.*

1. **Use the e-guide to calculate the Mayo score**

*Using the mayo calculator, you have assessed him as having moderate disease.*

1. **What is recommended for his medical therapy?**

*His oral 5-ASA is increased to 4g daily and he is started on rectal therapy. The response to treatment is reassessed in 4 weeks and he has not had any significant improvement in his symptoms.*

1. **What is the next step in his medical therapy?**

*You reassess his response to treatment over the following weeks with his symptoms resolving and achieving a steroid induced remission. His steroid is weaned and stopped over the next 2 months when he represents with return of his previous symptoms.*

*His stool culture is negative for clostridium difficile and his inflammatory markers are raised. You perform a flexible sigmoidoscopy which finds moderate-severe disease (Mayo score 8 – with loss of vascular pattern, erythema and erosions) at least to the splenic flexure.*

1. **What medical therapy is recommended next?**

*He is commenced on infliximab and a thiopurine and responds to treatment. You would like to plan what monitoring he will require for his thiopurine.*

1. **What thiopurine monitoring is suggested for the next 3 months?**

*18 months later, he sees you in clinic and would like to discuss stopping the infliximab. He has remained well on combination therapy since its commencement.*

1. **Your patient asks what is the risk of relapse within the first year of stopping infliximab?**

*Can you find the information on the e-guide regarding this?*

**Final Questions**

1. How did you find the process of using the e-guide?
2. What were the most helpful aspects of the e-guide?
3. What were the least helpful aspects?
4. Do you think anything should be changed with the e-guide?
5. What is your opinion of the recommendations given? Do you agree with them?
6. What would make a tool like this feasible to use in your everyday clinical practice?
