# Supplementary material for: Is the European Crohn’s and Colitis organisation (ECCO) e-guide an acceptable and feasible tool for increasing gastroenterologists’ guideline adherence? A mixed methods evaluation
Source: BMC Med Educ. 2024 May 13;24:529. doi: 10.1186/s12909-024-05540-w (PMC11092016; doi:10.1186/s12909-024-05540-w)
Supplement: Supplementary file 2 — Supplementary Material 2 [file 12909_2024_5540_MOESM2_ESM.docx]

Supplementary material Appendix C - Coding sheet summary

| **Theme** | **Code** | **Count** | **Illustrative quote** |
| --- | --- | --- | --- |
| **Accessibility** | Computer and internet access important | 6 | You mainly want to have an easy access on the phone or on the computer (ID1) |
|  | Mobile app increases use | 5 | I think if you can make it in an app form, on my phone, it would be a lot better. (ID11) |
|  | Hospital internet access limits usability | 2 | And then some of the websites I want to go to, the hospital intranet does not necessarily allow me to access rather than…. So for those reasons, I prefer on my phone. (ID11) |
|  | External website links cumbersome | 1 | It may make it a bit more cumbersome (ID1) |
| **Content Satisfaction** | Agree with recommendations | 11 | broadly speaking, I would agree with the guidelines, (ID12) |
|  | Disagreed with recommendations | 7 | I just don’t think if someone had moderate disease, give him next to add two grams of mesalazine – I definitely didn’t agree with that, (ID10) |
|  | Would use e-guide again | 7 | I will use this actually. (ID6) |
|  | Algorithms useful | 6 | I think the algorithm was very useful especially if we’re time poor. The other thing I liked about the algorithm is it’s not just like a simple picture, it's interactive, (ID1) |
|  | Algorithms unhelpful | 5 | but it does worry me that people try to follow these strictly and I think you can make the wrong decisions and it’s not as simple….So I actually find them quite useless. (ID12) |
|  | More management information needed | 5 | So just click that and then tell me how to do it. What is the evidence behind it. Rather than me having to search for it. (ID5) |
|  | PBS limits applicability in Australia | 5 | The only thing is if you have someone who’s had 5-ASA, a little period on steroids, and then flaring just when you stop them, and then just going straight to biologics. The only issue with that is I guess the website – if there was an Australian version, we’d have to take into PBS and Medicare and what’s available. (ID15) |
|  | Video needs additional information | 5 | Yeah. The video is useless without someone telling you about what they’re doing, (ID15) |
|  | Categorising patient challenging | 4 | Categorizing patients disease severity can be quite arbitrary like mild, moderate, severe…it's very difficult to categorize everything...The patient doesn't fit into one category (ID5) |
|  | Algorithms need improving | 3 | It’ll be hard to put in a one flowchart for everyone to follow, but I think those algorithms could be better. (ID12) |
|  | Information icon links helpful | 3 | so if I wanted to know more about a particular box, there’s that information icon which takes me to the corresponding ECCO statement which is good. (ID11) |
|  | e-guide checklists useful | 3 | The immunisation guide is a handy reminder of ticking the box to remind me of what to do with the immunisation. (ID13) |
|  | Recommendations not patient specific | 3 | I think in all these sort of flowchart algorithms, you don’t take into account the case of nuances of each people’s scenario. (ID12) |
|  | Patient resources useful | 2 | Well, I’ll probably use the patient handout stuff the most because I always find that really helpful. (ID3) |
|  | Links and reference useful | 2 | and having all the various links that can take you from one page to another topic seems to work fairly easily. (ID8) |
|  | Video useful | 2 | I did like the video. I thought that that would be useful, for instance, if I was operating in a resource sort of low area. I probably would be able to use that in my – I don’t dilate routinely, so I would use that. (ID9) |
|  | Stuck on frustrating aspects | 1 | So some aspects of it are a bit frustrating because you get stuck in a path and I don't think it necessarily makes sense sometimes. (ID5) |
|  | Don’t always follow recommendations | 1 | and sometimes clinically we don't follow those distinctly. (ID3) |
| **Familiarity with e-guide** | Easier use once familiar | 9 | So if you are familiar. I think it's easy to find the information you're looking for when you're with a patient...I think if I were to use it again. It probably may become a bit easier (ID1) |
|  | Unaware of e-guide | 4 | The problem is when I think is the general gastroenterologist would have no idea of this resource and they are the people who are most likely to benefit. (ID1) |
|  | Unfamiliar with e-guide | 4 | No. I haven’t used it much – (ID10) |
|  | Website training helpful | 3 | Your questions about actual disease scenarios and questions are actually a really nice way of learning about the website. So I did notice that there was a tour initially and often it's the clinical scenarios, and the very led questioning that made me find these extra things that I probably wouldn't have found otherwise. (ID7) |
|  | Promotion useful | 2 | I’d use it more...if it was just a bit more well known and it was, I don't know how accepted it is from an Australian point of view...I haven't heard many people talking about it from my own experience. (ID4) |
| **Guideline preference** | Printed guideline material useful | 3 | Look, if there was something that printed out. So often, with hep C stuff, you’d check their interactions online, and get it printed out. “Oh, this is the report” and just file that in the record or something. So, if there was a similar print out, you could just add easily to your record. It would be useful, (ID14) |
|  | Prefer ECCO guidelines | 2 | I use more the guidelines – actually, the ECCO guidelines rather than the e-Guide. (ID10) |
|  | Printed guidelines impractical | 1 | I can’t carry it everywhere. I can't carry it to, for example, the clinic in my rooms. (ID6) |
|  | Difficult to choose which guideline | 1 | As opposed to using it as a particular option to go to, you know, obviously there are American guidelines, etc. And trying to choose between the two obviously is a bit difficult, (ID4) |
| **Layout** | Concise specific information useful | 8 | and because many of the information here is actually quite packed with many things. So for example, about maintaining remission...So if I only have my mobile phone, I would think the letters would be very small, it probably needs to be summarised in a way...a more concise summary (ID6) |
|  | Layout helpful | 5 | It is quite intuitive to find where things are. I think the layout is good. (ID13) |
|  | Layout needs improvement | 5 | But I don’t like the homepage very much. Initially there’s all the tabs across the top, then the homepage kind of breaks down and just randomly across the page, there’s bits here and there. You kind of don’t get an idea of the whole – what’s on the whole website. The homepage could just be a link to – I don’t know. I’m not sure. But the homepage just seems like a mismatch of all those ones at the top whereas (ID5) |
|  | More visual information and less words needed | 4 | And I think visual aids are more helpful than pages and pages of text. (ID12) |
|  | Needs computer to visualise | 1 | Yeah. It’s not a problem at all (to visualise the e-guide) when I have the desktop and my desktop is very big. (ID6) |
| **Usefulness and Ease** | Easy to use | 8 | I think it answers very specific – it can help you answer very specific relevant clinical questions like the one you just told me. So in that way, it’s very, very helpful, and using data and not too much the actual answers that it gives you, the amount of information that it gives you, (ID10) |
|  | Time taken impacts use | 4 | I may have given up (looking for information) in a busy clinical setting. (ID12) |
|  | Useful if easily accessible | 3 | I mean, as long as there’s a way to quickly access things that you’re interested in, that would certainly help. I think if they’re distilled down into an easy place to find, that would be quite useful. So I think easy access, and being open access to anyone, (ID16) |
|  | Use at point of care | 3 | I’d probably use it in my point of care. (ID7) |
|  | Not user friendly | 2 | And if it was a little bit more user-friendly then you probably would use it a little bit more. (ID16) |
|  | Use in own time and point of care | 2 | I think I would use it both in front of me and use it in my own time. So for tricky cases, I would perhaps go home and think about what to do with some difficult patients, so if the answer is yes, I would use it for both away from patient and in front of patient. (ID13) |
|  | Useful for general gastroenterologist | 2 | because we practice a lot in this area and see a lot of patients with IBD, and look, I’m probably more familiar with the IBD literature than your general community gastroenterologist. So for me personally, probably less (useful), but I can see how it would potentially be very helpful for the generalist. (ID12) |
|  | Private practice use easy | 1 | Yeah, that’s right. I think it would be easier to refer to this in private practice. Yep. (ID9) |
|  | Use in own time | 1 | I’d probably use it in my point of care. (ID5) |
|  | Useful for IBD specialists | 1 | So definitely I think its good a difficult for an IBD specialist as well. So you've got your pregnancy stuff there, which I'm sure have it set of recommendations and you'll have your extra intestinal which, you know, people may not be so sure as well. I think it is. It does have a role for it, For the specialist as well. (ID1) |
|  | Feasible to use in practice | 1 | I think it’s definitely feasible now to use it in everyday practice (ID1) |
|  | No suggestions | 1 | I don't have any suggestions (for improvement) (ID13) |
|  | Therapeutic information useful | 1 | And I think it looks like there’s some really useful information about therapeutics that I don’t use commonly. So, that to me would be – that’s what I would use this for is fundamentally for the advanced therapeutics information and doing things like how to give information about stopping anti-TNF. That was a useful resource, so – yeah. (ID9) |
| **Perceived benefits** | Helpful for decision making | 12 | And I think areas that you're not as comfortable or deal like you know if you weren't sure about But I think for someone who doesn't treat Crohn’s or ulcerative colitis Often, I think it gives a nice step by step approach. Especially if you're not as comfortable don't have that much experience. It is good to be guided by that. (ID1) |
|  | Useful for patient discussion | 4 | I think it is helpful having some reference information that would be easy to reach for with patients like giving some easy to find data on specific clinical stuff like stopping therapy. I like the fact that – we didn’t go into it, but there are some illustrative diagrams there on risks of thiopurine... But I think having some easily accessible information that you can show your patients at the time and having the resource to print guides for them. (ID12) |
|  | eguide highlights knowledge gaps | 1 | but it does help to remind me of the gaps in knowledge (ID13) |
|  | Unsure if helpful | 1 | I don’t know that this would really help me in my clinical management (ID12) |
|  | Useful for simple tasks | 1 | I think the guide is useful for potentially simple stuff. I mean, the algorithms for what to do with a new patient or if someone is flaring are relatively simple (ID4) |
| **website navigation** | Difficult to navigate | 14 | I’m just moving here and here. There could be a search button here just if you wanted to type in something quickly and then it comes up with what you're looking for, like, if you didn't know. There's a lot of information tucked away. I wouldn't necessarily know the difference, like I probably wouldn't have gone to resources straight up and to try and find – I wouldn't have realised that the calculator, checklist, other slides, that they were there. (ID7) |
|  | Search function needs improvement | 12 | That search function seemed quite useless. Yeah. Yeah. So, it showed me nothing. So I think having a probably a better search function would be useful. (ID1) |
|  | More links needed | 4 | But it would be more useful if some of the topics were linked between the sub headings. Not having to go back to the menu that’s all. (ID3) |
|  | Easy navigation | 3 | I was surprised that you could actually find things without too much difficulty. (ID12) |
|  | Homepage needs improvement | 1 | It might be useful to – on your home page, give you an outline of what all the different categories are, like where you’d expect to find videos or where you’d expect to find calculators, ‘cause it’s up to a little bit of self-navigation versus having more of an introductory page. (ID8) |
|  |  |  |  |
